# Anterior cruciate ligament rehabilitation and return to sport in rock climbing athletes: a practical concept paper

**DOI:** 10.3389/fspor.2025.1580509

**Published:** 2025-09-25

**Authors:** Jared Vagy, Volker Schöffl, Julia Ohde, Wolf Petersen, Christoph Lutter

**Affiliations:** ^1^ Division of Biokinesiology and Physical Therapy, University of Southern California, Los Angeles, CA, United States; ^2^ Department of Orthopedic and Trauma Surgery, Sportsmedicine, Klinikum Bamberg, Germany; ^3^ Department of Trauma Surgery, Friedrich Alexander University Erlangen, Erlangen, Germany; ^4^ Section Wilderness Medicine, Department of Emergency Medicine, University of Colorado School of Medicine, Denver, CO, United States; ^5^ School of Clinical and Applied Sciences, Leeds Becket University, Leeds, United Kingdom; ^6^ University Medical Center Rostock, Rostock, Germany; ^7^ Department of Orthopedic Sports Medicine and Arthroscopic Surgery, Hessing Stiftung Augsburg, Augsburg, Germany; ^8^ Department of Orthopaedic and Trauma Surgery, Martin-Luther-Hospital, Berlin, Germany

**Keywords:** ACL, bouldering, rock climbing, protocol, return to sport

## Abstract

**Background:**

Acute ACL tears are becoming increasingly common among rock climbing athletes, particularly those who engage in bouldering.

**Hypothesis/purpose:**

The purpose of the paper was to develop a rehabilitation and return to sport protocol for rock climbing athletes after an anterior cruciate ligament reconstruction.

**Study design:**

The protocol is designed in 6 phases and emphasizes the importance of sport-specific postoperative rehabilitation protocols that address the unique needs of rock climbing athletes and stresses the need for sport-specific training prior to returning to full activity.

**Methods:**

The proposed rehabilitation protocol follows the stages of typical postoperative ACL protocols but with an emphasis on climbing-specific demands, such as eccentric landing mechanics and penduluming into a wall during lead climbing.

**Results:**

Climbers will be able to utilize the protocol to return to rock climbing.

**Conclusion:**

This paper highlights the need for further research on the topic and provides a foundation for future studies examining ACL tears in climbing athletes.

## Background

ACL tears are becoming an increasingly more common acute lower body injury in rock climbing, in particular within the discipline of bouldering (1). In a 4-year study of 71 patients with acute knee injuries in rock climbing, 7 ACL tears were reported bouldering, accounting for 9.1% of all acute lower body injuries (2). Although less common than medial meniscal tears and iliotibial band sprains, ACL injuries were the third most common acute knee injury reported in climbing (2). In contrast, ACL injuries in female soccer players, a sport with a reported high incidence of acute ACL ruptures have shown in studies to account for 6.6% of all acute lower body injuries (3). Although the increased prevalence of acute ACL injuries in bouldering compared to soccer may seem surprising, it shouldn't be, given the mechanical demands on the knee while falling during bouldering. Climbers often fall from height in uncontrolled positions, and it has been shown that insufficient landing control with immediate valgus loading is a contributing factor in the ACL injury mechanism (4).

In bouldering over ninety percent of injuries that caused at least a partial tear of the ACL resulted from a fall to the ground (1). Acute non-contact ACL tears falling from bouldering occur much differently when compared to court and field sports where the majority of ACL tears occur from a sudden deceleration prior to a change of direction (5). Boulderers must control high velocity landings on the ground from up to 3 meters while court and field athletes commonly need to control changes of direction at high speeds. Post-operative return-to-sport testing and training in court and field sports typically involve assessing distance, time, and movement performance in a variety of tasks, such as jogging, cutting, and multi-directional jumping. However, these assessments often overlook the eccentric component of landing from heights, such as the act of falling from a boulder problem and onto pad or uneven surface. Since the demands on the knee during bouldering and court/field sports are considerably different, it is important that post operative ACL protocols address the differences; both in sport-specific training and return to sport criteria. In addition to falls that occur during bouldering, the knee is also subjected to increased mechanical stress during lead falls in lead climbing. Lead climbing is a discipline in which the climber ascends while clipping the rope into protection points below them, which can result in longer falls that often involve a pendulum-like swing and impact with the wall (6). Therefore, a comprehensive post-operative return-to-sport training and testing program has been proposed for both bouldering and lead climbing. A recent publication on ACL injuries in climbing and bouldering named the following physical therapy goals: improvement of range of motion, muscle strengthening, and dynamic stability exercises progressing to functional performance (2). However, no detailed postoperative physical therapy protocol was suggested up to date. Further, no sport-specific return to sports test are available for climbing and bouldering yet; although they have been suggested for other climbing related injuries such as acute hamstring strains and growth plate fractures (7, 8). Therefore, we now aimed to introduce a postoperative rehabilitation protocol as well as sports specific return to climbing tests for rock climbing athletes following ACL repair/reconstruction.

## Post operative ACL reconstruction protocol for climbing athletes

The below is a post operative ACL reconstruction protocol for climbing specific rehabilitation. This protocol is a suggested guideline that can be used with the climber's post operative protocol. Phases are listed with estimated timeframes and criteria are given for phase progression. Rehabilitation progressions may vary based on the unique characteristics of each climber, the specific type of graft used during the operation, and any concurrent operations that may have been performed (i.e., meniscus repair). The protocol focuses on phase based rehabilitation with specific progression criteria rather than time based rehabilitation since there is considerable individual differences in biological healing, impairment resolution, neuromuscular control, functional skills, and psychological readiness with patients after ACL reconstruction (9). Suggested minimum timeframes for return to sport are provided to protect high functioning climbers from returning to sport too soon Table 1 (10).

**Table 1:** Post operative ACL reconstruction protocol for climbing athletes.

Phase	Intervention focus	Climbing specific activities	Phase goals: criteria for phase progression
Phase 1: (0–4 weeks) Early Post Operative	• Reduce pain • Decrease swelling • Initiate proprioceptive, balance, and neuromuscular re-education exercises • Gait train • Perform passive and active mobilizations of the tibiofemoral and patella femoral joint • Improve hip mobility (flexion and abduction) • Improve ankle mobility (dorsiflexion)	• Initiate seated or supine finger training • It is recommended that climbers wait until wound healing is complete to begin hangboarding	• Full knee extension AROM • Knee flexion PROM to 90°
Phase 2: (4–8 weeks) Late Post Operative	• Continue to improve range of motion • Improve conditioning of the knee extensors, knee flexors, ankle plantar flexors, hip abductors, and hip extensors • Increase challenge of proprioceptive, balance, and neuromuscular re-education exercises • Maintain/build finger strength and shoulder girdle strength for climbing • Initiate climbing movement specific isolated exercises • Improve upper extremity assisted step up ability	• Hanging shoulder girdle strength and endurance exercises • Initiation of hamstring strength heel hook training (if no contraindications) • Initiation of high step training starting at 10 cm	• Adapted Grant Foot Raise 80% • High Step Pull 0.5 meters×10 • Able to ascend 6 flights of stairs skipping 1 step each stride • Knee effusion ≤1+ with sweep test • Active range of motion knee flexion 90% of uninvolved side • Two weeks of single limb balance proprioceptive exercises with upper and lower extremity movement with the knee extended and flexed • Knee extensor, knee flexor, and hip abductor dynamometer MVIC > 70% of opposite leg
Phase 3: (8–16 weeks) Early Strengthening	• Initiate low risk vertical climbing movement • Improve strength of the knee extensors, knee flexors, ankle plantarflexors, hip abductors, and hip extensors	• Start top roping 3 grades below (VIII -> VI) estimated top rope ability on the UIAA scale (recommend to initiate after at least 12 weeks)	• Knee extensor, knee flexor, and hip abductor dynamometer MVIC 80% of uninvolved • At least 80% limb symmetry with single leg squat repetitions to 60 degrees of knee flexion with adequate control and quality • Y Balance anterior and posteromedial within 4 cm
Phase 4: (16–24 weeks) Late Strengthening	• Incorporate low risk lateral climbing movement • Regain quadruped weight bearing movement and coordination • Increase intensity and exercise selection challenge of the knee extensors, knee flexors, ankle plantarflexors, hip abductors, and hip extensors • Education on fall technique • Initiate beginner landing and plyometric training	• Submaximal boulder traversing no greater than.5 meters off the ground. Hinge stabilization brace recommended. • Top rope challenge progressed to 2 grades below (VIII -> VII+) top rope ability on the UIAA scale • Closed kinetic chain upper extremity and quadruped exercises mimicking climbing movements • Early stage landing training. Begin at low heights and progress up to 0.5 meters	• ACL-RSI ≥ 56 Points • Drop Hang score 90% • Lead Fall score 100% • Limb symmetry index of 85% with single hop for distance, 6-meter timed hop, triple hop for distance, and crossover hop for distance • Knee extensor, knee flexor, and hip abductor dynamometer MVIC 85% of uninvolved • Single limb leg press 85% of uninvolved
Phase 5: (24–36 weeks) Return to Sport	• Retrain proper landing mechanics (both boulder and lead climbing) • Strengthen and coordinate climbing specific movements • Progress to intermediate landing and plyometric training	• Start lead climbing 3 grades below (VIII -> VI) current top rope ability on the UIAA scale • Start vertical “no fall” bouldering two grades below (V8 -> V6) estimated bouldering ability • Drop knee training • Allowed to challenge climbing specific exercise technique practice on the wall in controlled situations with top rope. i.e., heel hook, high step, drop knee and progressing into utilizing those movements in bouldering and/or lead climbing • Landing training progression	• At least 9 months post operative • Maintains consistency with performing either strength training and plyometric training at least 3 times per week • Can demonstrate heel hooks, high steps, and drop knees while bouldering and/or lead climbing • Demonstrates ability to climb up to ability level for lead or bouldering • ACL RSI with a score of 75 points or greater • Limb symmetry index of ≥90% of uninvolved with hop testing • Knee extensor, knee flexor, and hip abductor MVIC ≥ 90% of uninvolved • Single limb leg press ≥ 90% of uninvolved
Phase 6: (36+ weeks) Return to Performance	• It is recommended that this phase begins no sooner than 36 weeks post operatively • Lower limb depth jumping at great heights • Advanced strengthening with proprioceptive challenge • Progress to advanced landing and plyometric training	• Competition lead climbing • Project lead climbing • Competition bouldering • (recommend to initiate after at least 12 months) • Projecting bouldering (recommend to avoid boulder problems over 3 meters until at least 12 months)	• The climber is recommend to continue performing therapeutic exercise up to 3 years after surgery to reduce the risk of reoccurrence of ACL injury

### Phase 1 (0–4 weeks)—early post operative

During the early postoperative phase, the interventions primarily revolve around pain management, edema control, range of motion, gait training, proprioception, and neuromuscular re-education. Alongside the standard postoperative interventions, it is important to prioritize early attention toward improving ankle and hip mobility. Research has highlighted the potential link between restrictions in dorsiflexion range of motion and an elevated risk of ACL injury due to altered landing mechanics and heightened ground reaction forces (11). Additionally, hip flexibility into abduction and flexion has been linked to rock climbing performance (12–14) and the greater amount of hip motion a climber can achieve, the less likely the knee joint will be stressed during end range motions.

Finger strength is a strong predictor for climbing performance (14) so it is important to keep the fingers conditioned for climbing while rehabilitating from an ACL surgery. Over 40 percent of rock-climbing injuries occur in the hand and fingers (15). Climbers are restricted from climbing during the early stages of rehabilitation, so are recommended to maintain their finger strength by performing seated or supine gripping exercises. These can be progressed to standing and using a portable fingerboard at their side. Once the climber has demonstrated appropriate wound healing and has demonstrated that they are comfortable hanging, they can initiate fingerboard training. The fingerboard is suggested to be mounted at a height that allows the climber to reach without lifting their legs off the ground. The proposed criteria for advancing to the next phase include achieving full active range of motion of knee extension and passive range of motion of knee flexion up to 90 degrees.

## Phase 2 (4–8 weeks)—late post operative

During the late postoperative phase the climber continues to improve range of motion and begins conditioning of the knee extensors, knee flexors, ankle plantar flexors, hip abductors, and hip extensors and increases the challenge of the proprioceptive, balance, and neuromuscular re-education exercises (16).

Shoulder girdle muscle endurance are a strong predictor for climbing performance (14) and the shoulder is second most injured climbing body region (17), so it is important to keep the upper body conditioned for climbing. During this phase, climbers are encouraged to maintain upper body endurance by performing pull-ups and 90-degree elbow flexion bent arm hangs. The pull-up bar is suggested to be mounted at a height that allows the climber to reach them without lifting their legs off the ground.

Climbers can begin focusing on early climbing specific lower extremity flexion and extension activities such as conditioning the hamstrings to pull (for a heel hook position) and conditioning the quadriceps and hip extensors to push (stepping up into a high step).

**Figure 1:**
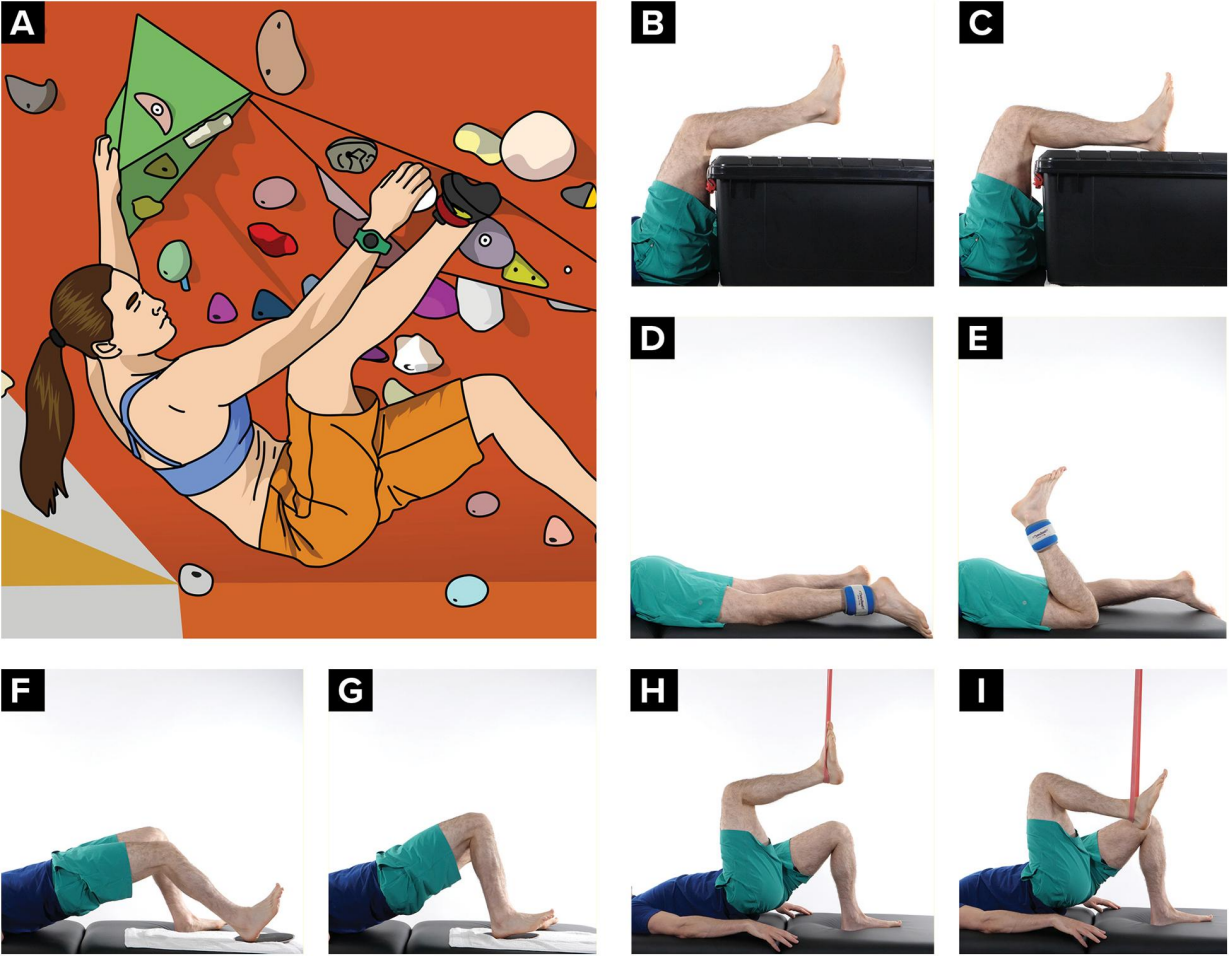
Heel hook progressive exercises. **(A)** Climber using a heel hook, **(B,C)** Hamstring exercise with hip flexed, **(D,E)** Hamstring exercise with hip extended, **(F,G)** Hamstring exercise in a double limb bridge, **(H,I)** Hamstring exercise in a single limb bridge.

A heel hook uses the lower extremity in a pulling fashion to help stabilize a climber, prevent extraneous body movement, or pull them further up the wall. The posterior aspect of the heel creates pressure and friction on a hold (Figure 1A) (18). This movement places significant force on the hamstrings and often places the climber's lower extremity in a position of external rotation. Since the hamstring provides control of tibial anterior translation, it is important to initiate hamstring strengthening early in the rehabilitation process (unless contraindicated until later with surgical precautions or a hamstring autograft). Exercises should begin with low velocity hamstring strengthening and progress to high load strengthening and functional reconditioning (7). In the early stages a climber can start with small range active knee flexion with hip flexion (Figures 1B,C) and then progress toward prone weighted full range knee flexion (Figures 1D,E). In the later stages of recovery, exercises can be progressed toward double limb bridging with slider (or exercise ball) curls (Figures 1F,G) and single limb bridging with resistance band curls (Figures 1H,I). The climber is encouraged to vary their hip position in the frontal plane (abduction and adduction), their ankle position in the sagittal plane (plantarflexion and dorsiflexion), and their tibial position in the transverse plane (medial rotation and lateral rotation) to mimic the positions while heel hooking on the rock wall.

**Figure 2:**
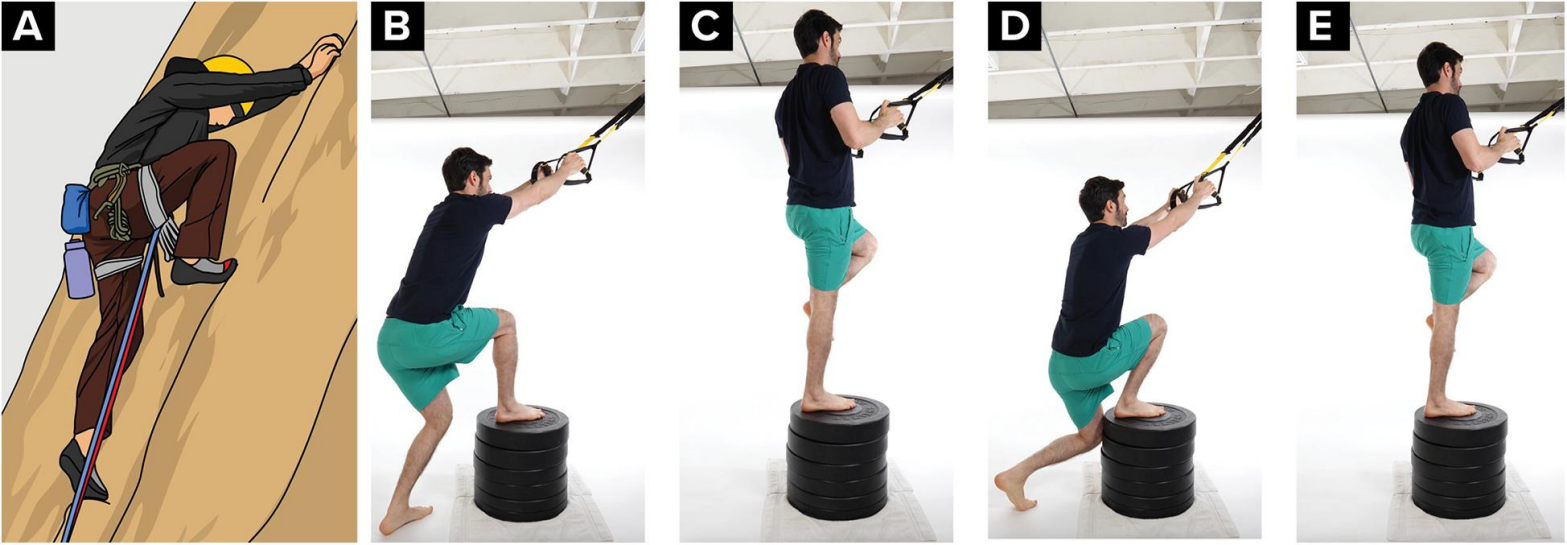
High step progressive exercises. **(A)** Climber performing a high step, **(B,C)** climber performing a high step and pull exercise with hip and knee flexed to 90 degrees in the starting position, **(D,E)** climber performing the exercise with greater amounts of hip and knee flexion in the starting position.

A high step is used during climbing when there is a high foothold that a climber needs to place their foot on to so that they can make the next move. A high step requires adequate ipsilateral hip flexion and abduction, and contralateral hip extension. To simulate the demands on the knee during a high step maneuver the climber is encouraged to use upper extremity support with suspension straps and portable fingerboards attached and then vary the challenge (Figure 2). The climber can start at a step of 10 centimeters and progress up to 0.5 meters.

**Figure 3:**
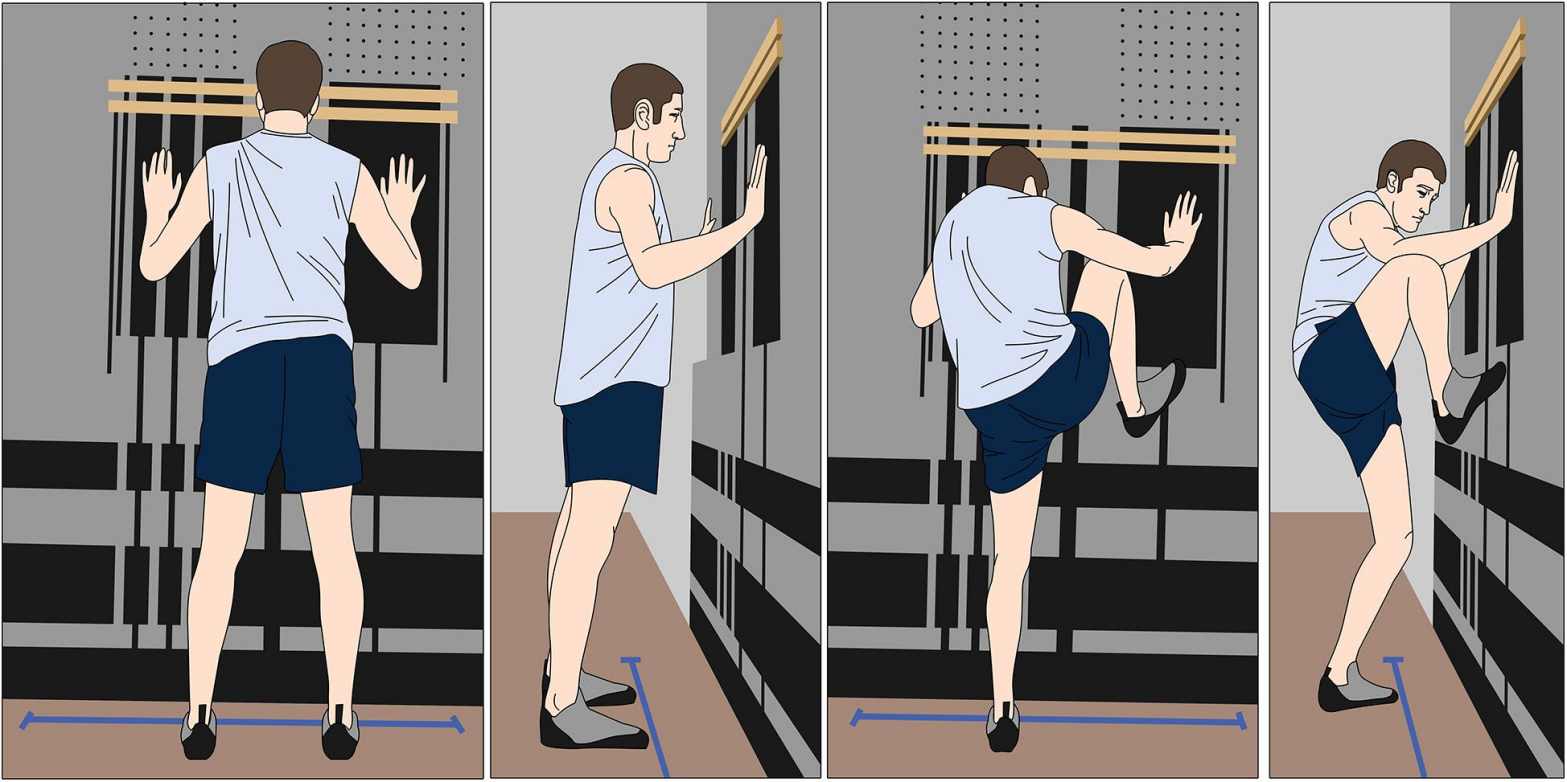
Drop knee progressive exercises. Adapted grant foot raise performed by a climber.

The suggested criteria for progressing to the next phase include the knee effusion ≤1+ with the sweep test, active knee flexion range of motion at 90% of the uninvolved side, two weeks of single-limb balance proprioceptive exercises involving upper and lower extremity movements with both extended and flexed knees, and knee extensor, knee flexor, and hip abductor dynamometer maximum voluntary isometric contraction (MVIC) measurements exceeding 70% of the unaffected side. Additionally, the climber needs to demonstrate the capability to execute 10 repetitions of a high step pull at a height of 0.5 meters. However, it is important to consider the possibility that a climber might rely excessively on their upper extremities while performing the high step pull. To address this potential concern, they must also exhibit the proficiency to ascend 6 flights of stairs, skipping 1 step with each stride. This stair-climbing test aims to replicate the uncompensated strength of the lower extremity, simulating the approximate vertical gain encountered during an indoor climbing route. Furthermore, a standardized assessment known as the Adapted Grant Foot Raise is recommended to evaluate high step mobility, a critical aspect for determining the climber's readiness to progress to the subsequent phase of top rope climbing (13). In this evaluation, the climber positions themselves 23 centimeters away from a wall, places their hands on the wall beyond shoulder width, and elevates their foot using a sliding motion to attain maximal hip flexion. The resulting height reached is measured, and it is expected that the achieved height falls within 80% of the maximal height on each side (Figure 3).

## Phase 3 (8–16 weeks)—early strengthening

The climber focuses on improving the strength of various muscle groups, including knee extensors, knee flexors, ankle plantar flexors, hip abductors, and hip extensors. Once the climber has achieved the milestones outlined in phase two and are minimum of 12 weeks post operative, clearance can be granted for climbing on vertical rock surfaces (where high stepping is frequently required), particularly on low-risk overhang routes secured by a top rope at a difficulty of three grades below (VIII -> VI) the climber's estimated top rope ability on the International Union of Alpine Associations (UIAA) scale. Climbers are recommended to use a hinged functional brace when they first start top rope climbing. The choice of beginning with overhang climbing before transitioning to vertical climbing aims to reduce the likelihood of foot entanglement with the rock wall in the event of a fall or an awkward step.

The suggested criteria to progress to the next phase are knee extensor, knee flexor, and hip abductor MVIC within 80% on dynamometry, and at least 80% limb symmetry with adequate motor control (defined as absence of trunk lean, knee valgus, and loss of balance) during single leg squats to 60 degrees of knee flexion. Previous research has indicated that individuals who have undergone ACL reconstruction who exhibit Y Balance Test deficits exceeding 4 cm are less likely to attain a limb symmetry index of 90% during hop testing (19). Considering these findings, an additional criterion of a Y balance test within a 4 cm range should be incorporated into the phase progression criteria. This criterion holds particular significance, given the next phase's clearance for boulder traversing—an activity demanding dynamic single-leg balance and outward reach capabilities.

## Phase 4 (16–24 weeks)—late strengthening

**Figure 4:**
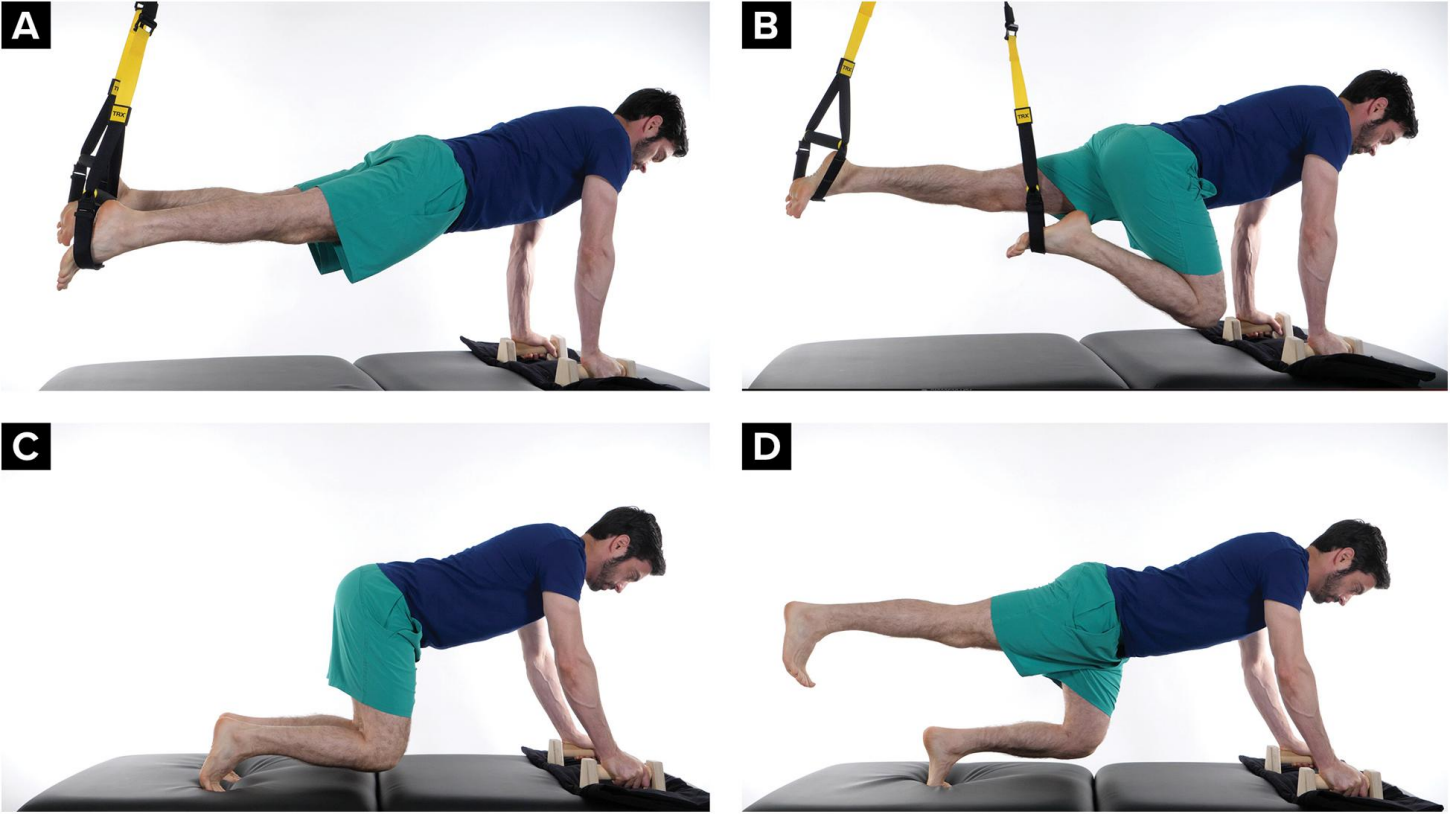
Closed kinetic chain upper extremity and quadruped exercises mimicking climbing movements **(A,B)** suspension training in a plank position with lower extremity movement, **(C,D)** quadruped bear position with lower body movement.

In this phase, the climber elevates the intensity and diversifies the selection of exercises to increase the demands on the knee extensors, knee flexors, ankle plantar flexors, hip abductors, and hip extensors. It is recommended to perform closed kinetic chain upper extremity exercises targeting the shoulder girdle and adjacent musculature with suspension equipment and weight bearing quadruped exercises that mimic rock climbing movement. Suspension training exercises have been shown to have greater levels of muscle activation (measured via electromyographic signal intensity) when compared to floor exercises (Figures 4A,B) (20) which is a benefit in rock climbing movements that require high levels of muscular demand. Quadruped exercises such as bear birddog mimic the cross-body movements (left arm reach, right leg extension) that are similar to climbing (Figures 4C,D).

**Figure 5:**
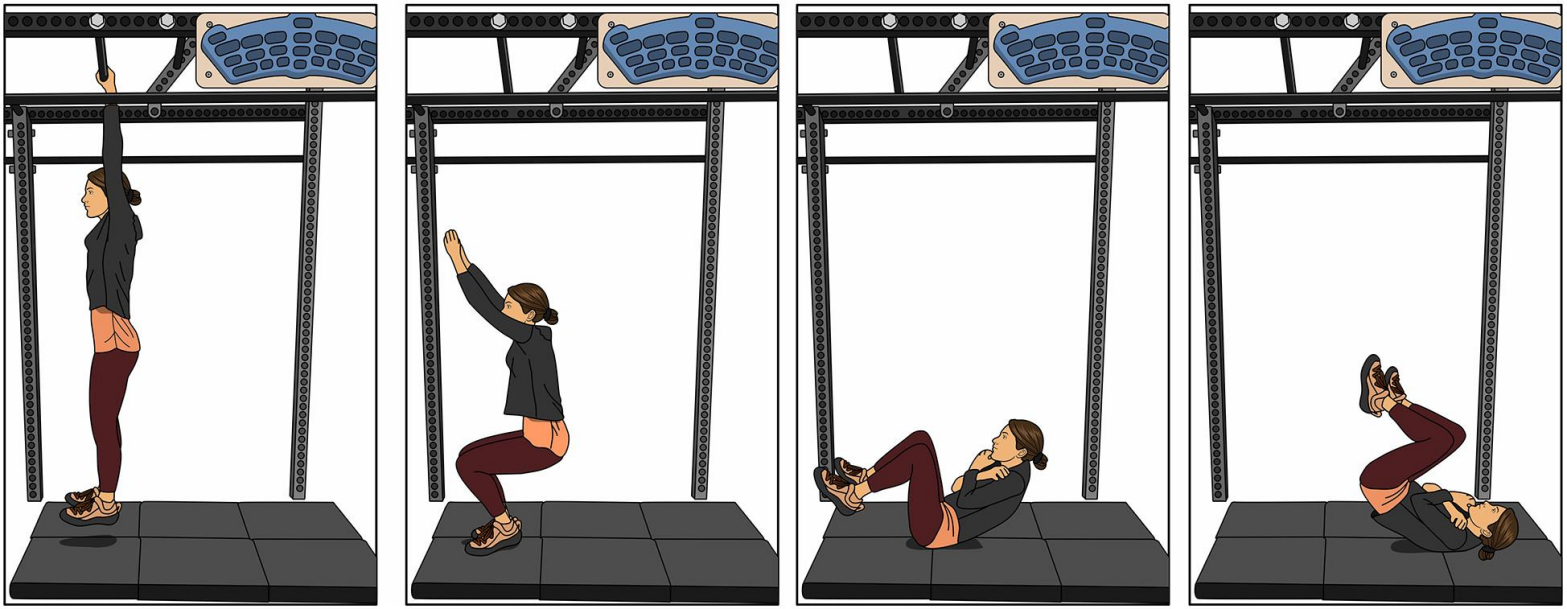
Image of a climber performing drop hang testing and training.

Furthermore, the climber is recommended to initiate beginner plyometric jumps and landing training. This includes “drop hang training” where the climber hangs from a pull-up bar (or steps off from a box), releases their grip, and lands into a 90-degree knee flexion angle squat (Figure 5). Drop hang training begins at a height of 10 centimeters at the start of the phase and progresses up to 0.5 meters by the end of the phase. Foot positions can vary to train the variation during boulder landing. This “drop hang training” will eventually utilize a standardized scoring criteria that a climber will need to achieve progress to the next phase (Table 2).

**Table 2:** Drop hang scoring system (five drops per level).

Level	Description	
Level 1	Release grip and land on bouldering pad with both legs neutral	
Level 2	Release grip and land on bouldering pad with operative leg in front of non-operative leg	
Level 3	Release grip and land on bouldering pad with non-operative leg in front of operative leg	
Criteria	Yes (1)	No (0)
Knee flexion angle is ≥90°		
Performs repetitions without dynamic knee valgus (patella falls medial to the great toe)		
Lands with the correct foot orientation		
Rolls onto their backside		

Total Points: _______/20.

The climber is cleared for boulder traversing or treadwall climbing in this phase. Boulder traversing is lateral climbing at a height of 0.5 meters from the ground. Climbers should take into consideration the ACL stresses of tibial external rotation during boulder traversing (21) and are recommended to use a hinged functional brace both as a psychological reminder to not step off or fall on the affected leg and to provide additional stability to the healing ligament. Previous studies have shown the benefits of early-stage neuromuscular control exercises in the rehabilitation of post operative ACL injuries (22). So as long as the climber maintains a safe boulder traverse height, wears a brace, and is trained to step off the boulder with their non-surgical limb, submaximal boulder traversing is considered safe.

**Figure 6:**
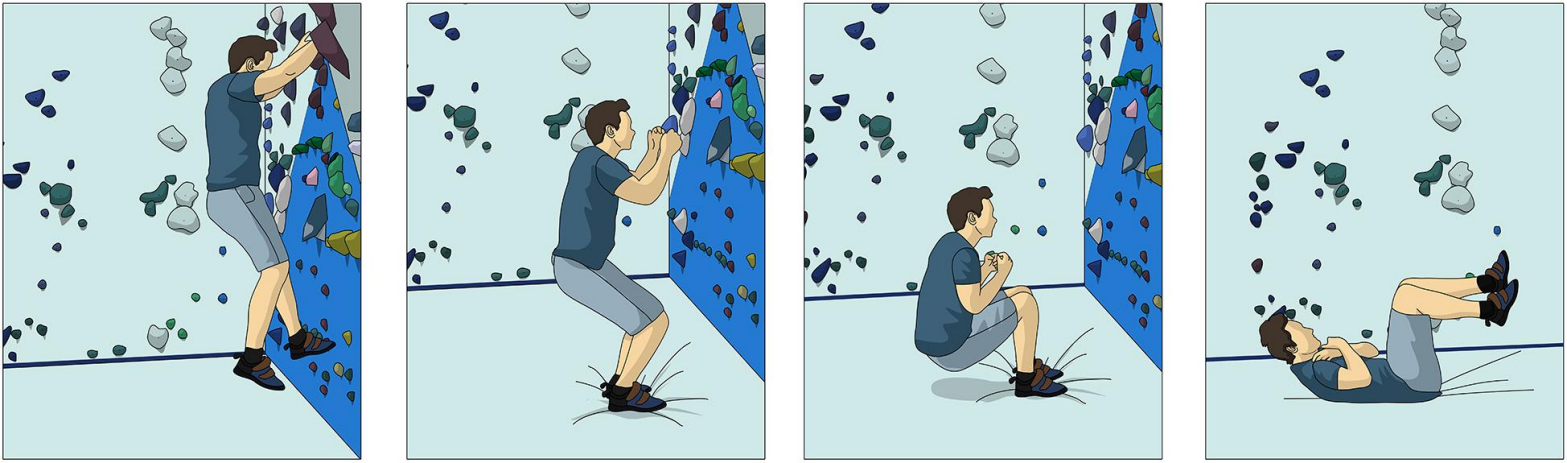
Backward falling from a boulder problem.

Additionally, in the case the climber does fall onto their feet, they should be taught how to help attenuate the force on the knee with the four steps for falling on their back; stay relaxed, bend knees and arms, tuck arms in, and roll back (Figure 6).

**Figure 7:**
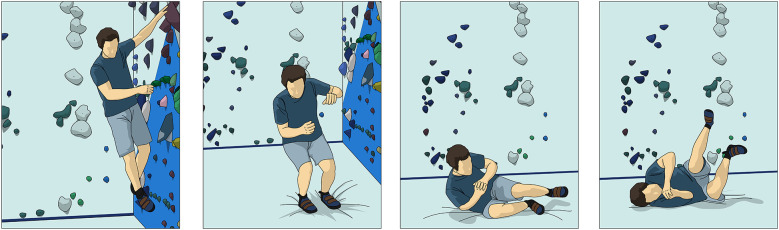
Sideways falling from a boulder problem.

The climber will also be educated on how fall on their side. The climber will be educated if they fall toward one side to look at the landing zone, bend knees and arms, tuck arms in, and let the pads do the work (Figure 7). Climbers should be cautioned to not lock their knees while landing and to not use their upper extremity to break the fall.

Once the climber has acknowledged they understand fall technique, they can be coached from a squatting position how to control a roll onto their back and side in the case they fall off the wall and are unable to land on their back while boulder traversing.

The suggested criteria to progress to the next phase (return to climbing) includes the following: ≥56 points on the ACL return to sport after injury, 90% score on the 18 inch (0.5 meter) Drop Hang Test, 100% score on the Lead Fall Test, and a limb symmetry index of 85% with single hop for distance, 6-meter timed hop, triple hop for distance, and crossover hop for distance. Additionally, knee extensor, knee flexor, and hip abductor dynamometer peak force within 85%, single limb leg press at 85% of the non-surgical leg. If the climber meets these criteria and has reached a minimum of 24 weeks post-operation, they are eligible to progress to the next phase, which encompasses “no fall” bouldering and lead climbing.

## ACL—return to sport after injury (ACL-RSI)

It has been shown that athletes who have undergone ACL reconstruction surgery have high levels of fear-avoidance and anxiety with returning to sport (23). If a climber is fearful when returning to climbing and the psychological components are not addressed beforehand, they may overgrip, poorly sequence movements, or struggle on moves they are physically capable of performing. In climbing, this can increase stress on the operative leg or result in a catastrophic fall. It is for these reasons that psychological testing should be performed for all climbing athletes to determine their readiness to return to sport. It is recommended that climbers achieve a score of ≥56 points on the ACL RSI before proceeding with Drop Hang Testing, Lead Fall Testing, and advancing to the subsequent phase to return back to boulder and lead climbing (24).

## Drop hang scoring

Research has shown that hop testing, such as single hop for distance, 6-meter timed hop, triple hop for distance, and crossover hop for distance, are reliable and valid performance-based outcome measures for return to sport after ACL reconstruction in court/field sports (25). Additionally, tests involving movements such as squatting, lateral bounding, jogging, and directional changes are also commonly used (26). However, although these tests provide reliable and valid performance-based outcome measures for returning patients after ACL reconstruction to court/field sports, they do not reproduce the demands needed to return back to bouldering.

The drop hang is used to determine a climber's readiness to fall safely from a boulder problem. The scoring uses a 20-point scale to grade the ability for the climber to land safely. The climber is recommended to use their climbing shoes to simulate the same environment that they would land if they fell from a boulder problem. The test requires a pull-up bar or rings and a soft-landing surface, commonly a bouldering crash pad. The climber starts by grasping the pull-up bar or rings with the elbows flexed or extended. The clinician will then adjust the rings or have the climber flex their elbows until their feet lift to 0.5 meters from the pad (Figure 5—at an increased height). If the set-up is difficult to adjust, a stable 0.5 meters surface to step backwards off can be used instead.

The climber will release their grip and land onto the pad five times while the clinician scores them based on four criteria: knee flexion angle, knee stability, accuracy of landing, and ability to attenuate shock by rolling onto their backside. Each category earns the climber 1 point for each repetition that meets the criteria.

The clinician will test the climber in three different levels: a neutral stance (level 1), operative leg in front of non-operative leg with the toe of the back foot aligned with the heel of the front foot (level 2) and non-operative leg in front of operative leg with the toe of the back foot aligned with the heel of the front foot (level 3). Climbers will start at level one and must score a minimum of 18 out of 20 points (90%) to advance to the next level (Table 2).

Prior to initiating drop hang testing, climbers should have advanced beyond the initial stage of landing training. This advancement entails performing box step-offs and systematically elevating the height until they have attained a sense of ease landing from 0.5 meters in a squat position in a variety of foot positions.

## Lead fall scoring

A lead fall test is used to determine the climber's readiness to return to lead climbing. It is scored on a 20-point scale and requires the climber to have access to a lead climbing route and a belaying partner who can catch their lead fall.

The climber is advised to begin with a graded progression of clipping and falling on overhung routes with the last quick draw at the level of the hip, knee, and then foot level. Once the climber can safely demonstrate this progression, they can progress to a pendulum fall on a vertical or slightly overhung terrain.

For each category in the scoring system, the climber receives 4 points if they meet the specified criteria. The climber must score 20/20 (100%) to pass the test. If there are pre-existing or other physical impairments, some leeway may need to be allowed for the test (Table 3).

**Table 3:** Lead fall scoring system.

Criteria	Yes (4)	No (0)
Knee flexion angle is ≥60°		
Hip flexion angle is ≥60°		
Demonstrates appropriate technique and safety considerations of identifying the fall zone		
Keeps the rope between the climber and the wall		
Exhales upon release and keeps a relaxed body		

Total Points: _______/20.

## Phase 5 (24–36 weeks)—return to sport

Returning to lead climbing and bouldering should be approached with caution because of the possible fall risk. Although the window to enter phase 5 starts at 6 months, many climbers don't achieve the needed milestones to return to climbing until closer to 9 months. Between 6 and 9 months post-operative, each month of delayed return to sport may decrease the risk of re-injury by 51% (10).

However, once the climber has achieved the necessary milestones and has entered this stage, they are cleared to lead climb 3 grades below (VIII -> VI) current top rope ability on the UIAA scale and are cleared to boulder 2 grades below their projected bouldering ability (V8 -> V6). The climber is cautioned to climb only boulder routes where they will not fall. The climber is encouraged to return to climbing first indoors in a controlled environment and then progress toward outdoor climbing.

**Figure 8:**
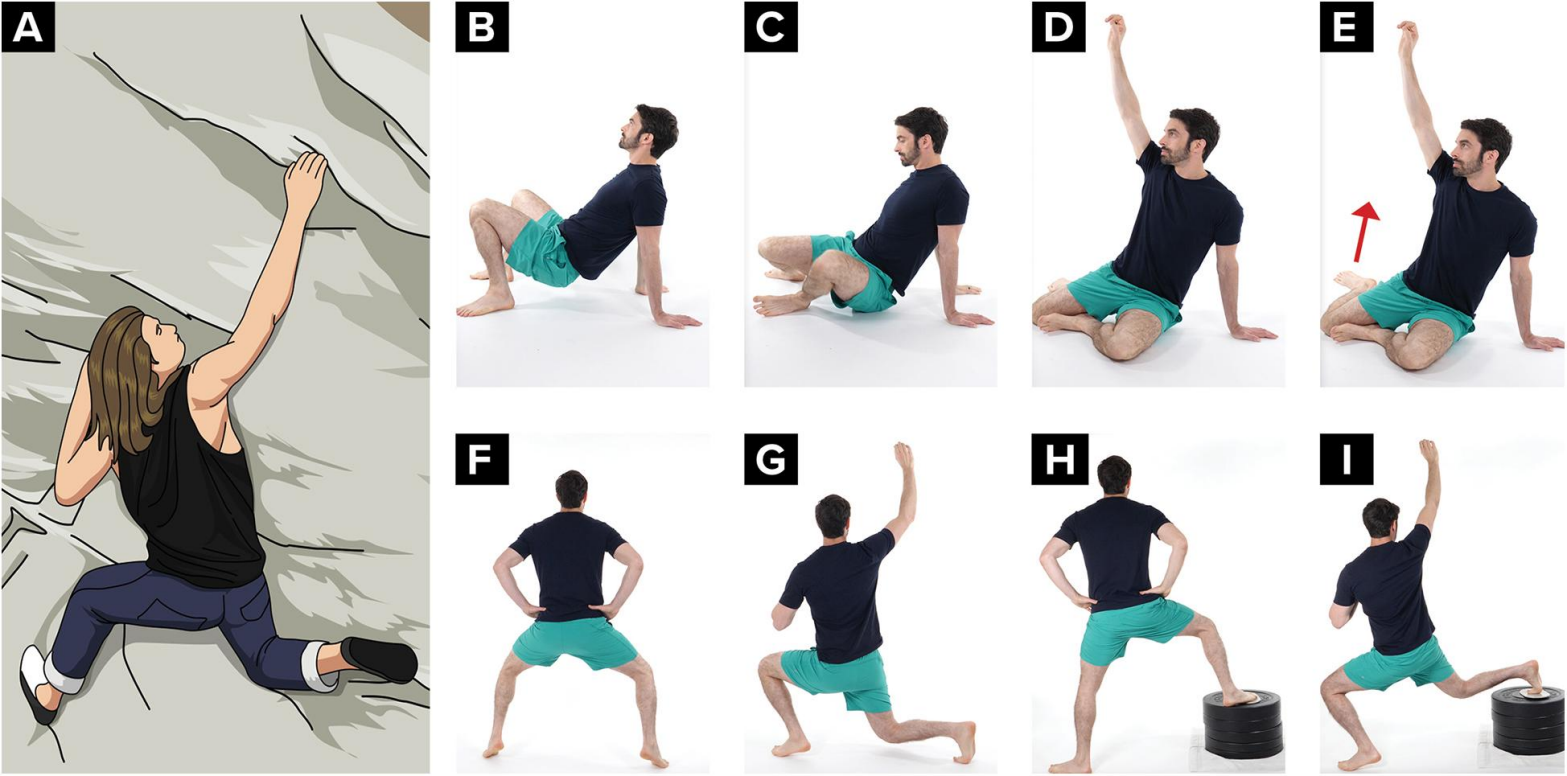
Drop knee progression from sitting, to standing, to an elevated foot **(A)** climber demonstrating a drop knee, **(B–E)** on the ground hip internal rotation exercise, **(F,G)** standing drop knee exercise, **(H,I)** standing drop knee exercise progression with foot on an elevated surface.

The climber is encouraged to begin training the knee to withstand the controlled valgus stresses that may occur during a climbing movement called a drop knee. A drop knee is used while climbing when there are two foot holds that need to be utilized with opposing foot pressure. The climber puts their weight on the outside of one foot, rotates their hip in toward the wall and lowers their knee; the opposite foot presses against an opposing hold for support. A drop knee requires adequate rectus femoris muscle length and adequate hip internal range of motion. A drop knee can be trained starting on the ground with the hip and knee bend to 90 degrees and then progressed into squat standing, and then with the foot on an elevated the surface (Figure 8).

Drop knees, heel hooks, high steps, and falls have all been shown to be the primary mechanism acute knee injuries (2). So in addition to adding drop knee specific exercise progressions, climbers are encouraged to begin slowly and conservatively utilizing drop knees, high steps, and heel hook progressions from maximum assistance to minimum assistance while climbing.

Progressing landing training in a variety of environments is an essential component of returning to boulder training. A heavy emphasis should be placed on controlled fall practice. This includes controlling the height, base of support, strategy, and landing surface, in addition to providing cueing to help attenuate shock and technique training to improve protective mechanisms while falling. Drop hang training can be challenged by increasing the height. The base of support can be progressed from wide, to narrow, with variations of symmetric/offset alignment and neutral/toed out foot position. Landing strategy can be progressed from a forward trunk lean to an upright trunk position. Starting with a forward trunk lean helps to distribute forces in the gluteal muscles, while progressing to a more upright trunk landing position helps to distribute forces to the knee extensor mechanism (27). Climbers are cued to land softly as this has been shown as an external verbal cue that helps decrease loads on the ACL when landing (28).

The suggested criteria to progress to the next phase are that the climber is least 9 months post operative, ACL RSI with a score of 75 points or greater, maintains consistency with performing either strength training and plyometric training at least 3 times per week, can demonstrate heel hooks, high steps, and drop knees while bouldering and/or lead climbing, and demonstrates the ability to climb up to ability level for lead or bouldering.

While much of this phase emphasizes movement specific loading, fall retraining, and psychological readiness, it is equally important to consider the broader physical and physiological demands of climbing. Although climbing is not typically classified as an aerobic sport, research has shown that both lead climbing and bouldering place meaningful demands on the cardiovascular and respiratory systems, including increased heart rate, elevated oxygen uptake, and tidal volume constraints (29, 30). Additionally, fatigue can negatively impact technique, motor control, and decision making, and has been shown to be a risk factor related to ACL injuries (31). Therefore, return to sport programming should incorporate simulated maximal effort climbing in safe, controlled environments, such as low to the ground bouldering and top rope scenarios for lead climbers, to prepare athletes for the intensity of full height bouldering and lead climbing.

## Phase 6 (36+ weeks)—return to performance

At this stage the climber's limb symmetry index with hop testing, MVIC, and single limb leg press should all be greater than or equal to 90% of the uninvolved leg. It is encouraged to continue performing therapeutic exercise up to 3 years after surgery to reduce the risk of reoccurrence of ACL injury. This includes advanced strengthening with proprioceptive challenge, advanced landing training, and advanced plyometric drills. Rehabilitation includes lower limb fall practice at great levels of challenge, plyometric training, and advanced strengthening with proprioceptive challenges. The climber works toward competition lead climbing and project lead climbing. It is recommended to begin competition and project bouldering no sooner than 12 months and to refrain from attempting boulder problems exceeding 3 meters in height.

## Conclusions

ACL tears are a growing concern in rock climbing, particularly in bouldering, and a comprehensive postoperative rehabilitation protocol with sport-specific return-to-sport testing is necessary to address the unique demands of the sport. Our research detailed a new rehabilitation protocol for climbers following ACL reconstruction, including return-to-sport tests tailored to the physical demands of both bouldering and lead climbing. These tests account for climbing-specific variables such as eccentric landing mechanics and pendulum-style falls during lead climbing. Early use of the protocol with individual climbers has shown promise (32). Further studies are needed to evaluate its safety and effectiveness.

## Data Availability

The original contributions presented in the study are included in the article, further inquiries can be directed to the corresponding author.
